# Radioactive Iodine Administration Is Associated with Persistent Related Symptoms in Patients with Differentiated Thyroid Cancer

**DOI:** 10.1155/2016/2586512

**Published:** 2016-10-27

**Authors:** Pablo Florenzano, Francisco J. Guarda, Rodrigo Jaimovich, Nicolás Droppelmann, Hernán González, José M. Domínguez

**Affiliations:** ^1^Department of Endocrinology, Faculty of Medicine, Pontificia Universidad Católica de Chile, Santiago, Chile; ^2^Department of Nuclear Medicine, Faculty of Medicine, Pontificia Universidad Católica de Chile, Santiago, Chile; ^3^Department of Surgery, Faculty of Medicine, Pontificia Universidad Católica de Chile, Santiago, Chile

## Abstract

*Context*. Radioiodine (RAI) administration has adverse effects in patients treated for thyroid cancer (DTC), but there is scarce information regarding their intensity and duration.* Objective*. To evaluate frequency and intensity of early and late RAI-related symptoms in patients with DTC.* Design*. Observational prospective study.* Patients*. DTC patients who underwent thyroidectomy, with or without RAI.* Measurements*. Patients answered 2 surveys: (1) from 0 to 6 months and (2) between 6 and 18 months after initial treatment.* Results*. 110 patients answered the first survey and 61 both. Nearly 80 percent received RAI. Among early symptoms, periorbital edema, excessive tearing, salivary gland disturbances, dry mouth, taste disorders, and nausea were more frequent and intense among RAI patients. Regarding late symptoms, periorbital edema, salivary gland pain and swelling, and dry mouth were more frequent and intense in RAI patients. Frequency and intensity of adverse effects were not different between low and high RAI doses (50 versus ≥100 mCi).* Conclusion*. RAI-related symptoms are frequent and usually persist after 6 months of administration, even when low doses are given. This finding must be considered when deciding RAI administration, especially in low risk patients, among whom RAI benefit is controversial.

## 1. Introduction

The initial treatment of Differentiated Thyroid Cancer (DTC) includes surgery and selective administration of radioactive iodine (RAI) [1]. RAI has shown to decrease mortality and recurrence in high risk patients, and it is considered an important component of the treatment in invasive or metastatic disease [1, 2]. However, the benefit of RAI in low risk patients has not been fully supported by evidence [3–6] and current guidelines recommend an individualized approach for its indication [1, 7, 8].

RAI is not harmless. Several cohorts of patients treated with RAI have shown an increased risk of secondary malignancies even when RAI was used to treat low risk patients [9, 10]. More frequently, RAI can impact nonthyroidal tissues that express sodium iodide symporter (NIS), including the salivary glands and lachrymal system [11]. Salivary glands dysfunction occurs with an incidence that varies from 5 to 86% [12]. Dysfunction may appear as salivary gland pain, taste impairment such as hypogeusia, also xerostomia, sialolithiasis, dental caries, stomatitis, salivary gland or oral infections, facial nerve damage, and even salivary gland neoplasia [12]. These side effects may be persistent in nearly 7% of patients and profoundly impair patient quality of life (QoL) [13, 14]. Lachrymal system side effects can be manifested as epiphora, xerophthalmia, and recurrent or chronic conjunctivitis [15]. The time course and resolution of lacrimal effects are not completely known [15].

Although tissues in the nasal cavity do not express NIS, RAI may be transported nonspecifically from the lachrymal system into the nasal ducts [12]. Several nasal complaints have been documented including nasal pain, nasal tenderness, bloody discharge, and dry nose, with a frequency up to 10.5% [16]. As for lachrymal complaints, little information has been published regarding the duration of nasal symptoms.

Despite the uncertainty regarding the benefit of RAI and the potential adverse effects related to its use, recent evidence showed an increase in the use of RAI in patients with DTC in the last two decades, including those with low risk of recurrence [17, 18].

The objective of this study was to evaluate the frequency and intensity of early and late RAI related symptoms in patients with DTC.

## 2. Methods

We designed an observational, prospective study to evaluate and compare the frequency and intensity of early and late adverse effects associated with RAI administration in patients with DTC treated with total thyroidectomy. Our study protocol was approved by the Research Ethics Committee of our Institution, and all patients accepted to participate providing their informed consent.

Patients over 18 years of age with DTC, surgically treated in our institution between January and August 2013, were consecutively invited to answer 2 online surveys on 12 adverse effects associated with RAI administration. These surveys were created by our team based on information published in literature regarding RAI related symptoms [12, 13, 15, 16]. Early RAI related symptoms were assessed within 6 months after initial treatment (considering RAI administration date in patients who received RAI or surgery date in those who did not receive RAI). Late RAI related symptoms were assessed at least 6 months after initial treatment. Intensity of symptoms was evaluated with a Likert-like scale from 0 (none) to 5 (severe). For the purpose of data analysis, we determined four categories of intensity: 0 (inexistent), 1-2 (mild), 3-4 (moderate), and 5 (severe). The detail of the survey is shown in Table 1 in Supplementary Material available online at http://dx.doi.org/10.1155/2016/2586512.

All patients underwent total thyroidectomy. Lymph node dissection was performed in those in whom preoperative ultrasound, fine needle aspiration biopsy, or intraoperative findings suggested lymph node metastases. No prophylactic neck dissections were performed. We used fixed RAI doses based on the extent of initial disease. The administration of RAI and its dose were decided by the attending physician following the ATA and local guidelines, considering postoperative stimulated thyroglobulin [7, 19]. A low-iodine diet was prescribed 2 weeks before RAI administration and all patients received RAI at least 3 weeks after thyroid hormone withdrawal. A TSH above 30 mIU/L was certified before RAI administration as well as a negative pregnancy test in fertile women. Thyroid hormone replacement therapy was started 48 hours after RAI administration and all patients had TSH levels below 1.0 *μ*UI/mL six weeks after starting treatment. A posttherapy whole body scan (WBS) was performed five to seven days after RAI administration. We defined RAI doses as low and high when ≤50 mCi (1850 MBq) and ≥100 mCi (3700 MBq) were administered, respectively.

Considering the results of preoperative ultrasound, intraoperative findings, postsurgical histology, and WBS when RAI was administered, patients were risk-stratified using the 7th edition of the AJCC/UICC staging system (stage I, II, III, or IV) and the ATA risk of recurrence stratification system (low, intermediate, or high risk of recurrence) [19].

Categorical variables are expressed as number and frequencies; continuous variables are expressed as mean ± SD or median (range), when appropriate. Categorical comparisons were made using chi-square testing with Fischer's exact test when appropriate. The comparison between frequencies of early and late RAI related symptoms was tested with McNemar's test. Analysis was performed using SPSS software (version 15.0.0: SPSS, Inc., Chicago, IL) and Stata 12® (StataCorp, Collage Station, TX). *p* values < 0.05 were considered to be statistically significant.

## 3. Results

One hundred and ten patients answered the first survey and 61 the second one regarding early and late RAI related symptoms, respectively. The demographics, clinical features, risk stratification, and final outcomes included in this study are presented in Table 1. As expected, most patients were female (91.8%) and had low or intermediate DTC risk of recurrence based on the ATA 2009 risk stratification system.

All patients underwent total thyroidectomy, and 26 (23.6%) also underwent lymph node dissection. Of the total cohort, 86 (78.2%) patients received RAI: 38 (63.3%) in the low and 48 (96%) in the intermediate risk of recurrence patients. Patients received a median dose of 75 mCi (30–150 mCi) (median 2775 MBq; range 1110–5550 MBq). Patients who received RAI were similar to those not receiving RAI regarding age, gender, type of surgery, and time to surveys.

### 3.1. Early RAI Related Symptoms

**Figure 1:**
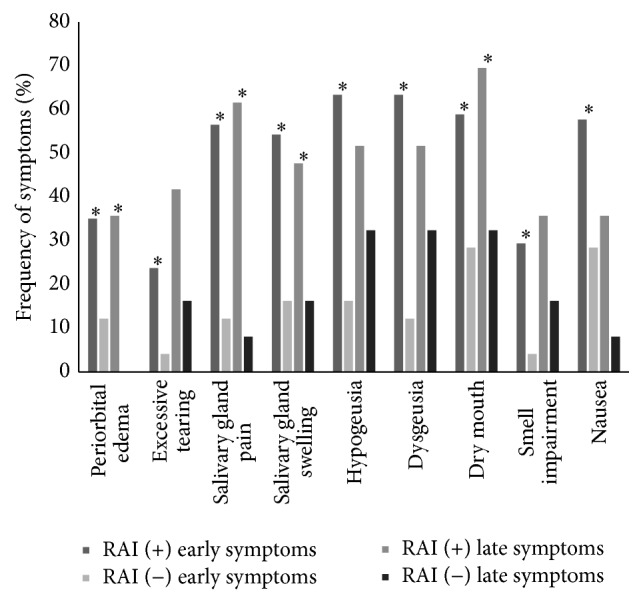
Frequency of early and late RAI related symptoms in patients with DTC treated with or without RAI. *∗* shows statistically significant differences (*p* < 0.05) between patients who received RAI and those who did not.

**Figure 2:**
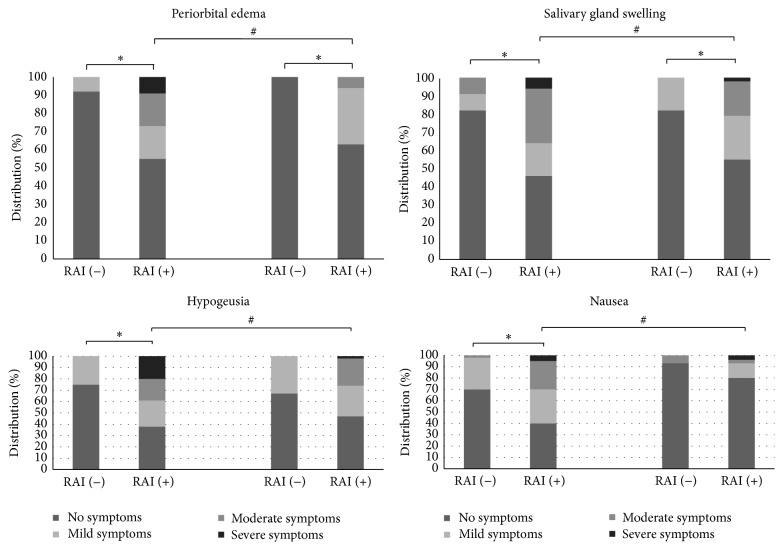
Distribution of intensity of early and late RAI related symptoms in patients with DTC treated with or without RAI. ^*∗*^Statistically significant difference in intensity of symptoms among patients who did and those who did not receive RAI. ^#^Statistically significant difference in intensity of symptoms among patients who received RAI between the first and the second survey.

The first survey was answered at a median of 2.5 (0.5–5.8) months after initial treatment. The following RAI related symptoms were more frequent among patients who received RAI: periorbital edema (36% versus 12.5%; *p* = 0.027), excessive tearing (24.4 versus 4.2: *p* = 0.028), salivary gland swelling (55.8 versus 16.7; *p* = 0.001), salivary gland pain (58.1 versus 12.5; *p* < 0.01), dry mouth (60.5 versus 29.2; *p* = 0.007), taste disorders (hypogeusia [65.1 versus 16.7: *p* < 0.01] and dysgeusia [65.1 versus 12.5: *p* < 0.01]), smell impairment (30.2 versus 4.2: *p* = 0.009), and nausea (59.3 versus 29.2: *p* = 0.009) (Figure 1). Intensity of symptoms was also significantly higher in patients receiving RAI treatment compared with those who did not (Figure 2).

**Figure 3:**
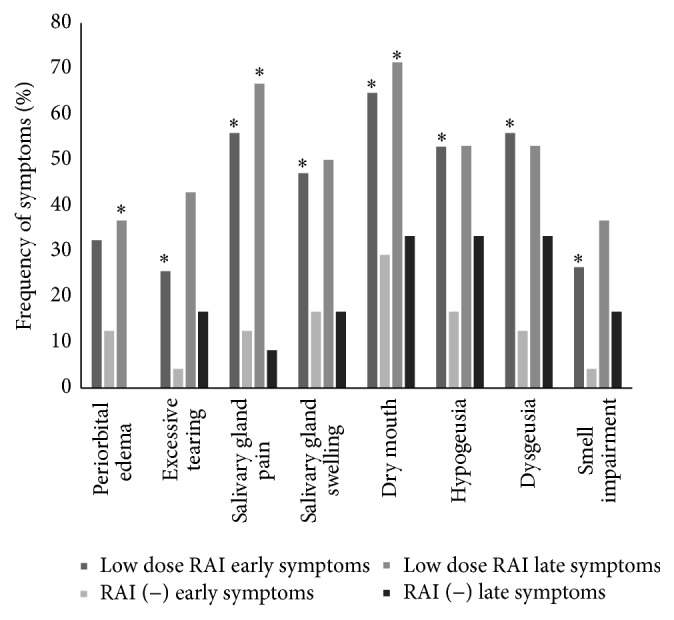
Frequency of early and late RAI related symptoms among patients who received low RAI dose (≤50 mCi) and those who did not. *∗* shows statistically significant differences (*p* < 0.05) between patients who received low RAI dose and those who did not.

Also, excessive tearing, salivary gland swelling and pain, dry mouth, and taste and smell disorders were more frequent in patients receiving low RAI doses (≤50 mCi) (1850 MBq) than in those who did not (Figure 3). When low and high RAI doses were compared, we found no differences neither in frequency nor in intensity of any of the studied RAI related symptoms.

### 3.2. Late RAI Related Symptoms

The second survey was answered at a median of 11.5 (6–18.6) months after initial treatment. The following RAI related symptoms were more frequent and intense among patients who received RAI: periorbital edema (36.7 versus 0: *p* = 0.012), salivary gland pain (63.3 versus 8.3: *p* = 0.001), salivary gland swelling (49 versus 16.7: *p* = 0.042), and dry mouth (71.4 versus 33.3: *p* = 0.014) (Figure 2). The same differences were found between low dose and no RAI administration (Figure 3). When low and high RAI doses were compared, we found no differences regarding the frequency or intensity of any of the studied symptoms.

Among those patients who received RAI, we found that epiphora and nausea were significantly less frequent in the second survey than in the first one. In terms of intensity, periorbital edema, salivary gland pain and swelling, taste disorders, and nausea were significantly less intense after six months of follow-up (Figure 1).

## 4. Discussion

In this study, we found a higher frequency of some early RAI related symptoms (periorbital edema, excessive tearing, salivary gland swelling and pain, dry mouth, taste and smell disorders, and nausea) among patients with DTC who received RAI than in those who did not. It is remarkable that, even after 6 months of initial treatment, periorbital edema, salivary gland pain and swelling, and dry mouth were persistently more frequent among patients who received RAI. It is also important to note that most of these RAI related symptoms were more intense among patients who received RAI.

In our series, most RAI related side effects and their intensity decreased 6 months after initial treatment. However, symptoms like excessive tearing, salivary gland pain, and dry mouth were more frequent, though less intense, 6 months after initial therapy. Although side effects usually decline in a longer follow-up period, the higher frequency and intensity of symptoms during the first year after RAI administration force us to take this information into account when considering RAI use in low risk DTC patients [13]. Nearly 60% of the low risk of recurrence patients of this cohort received RAI. We are aware that the latest evidence does not support RAI administration in this group of patients, but guidelines followed to treat these patients were more permissive regarding this issue [7, 19].

Literature is controversial regarding the relationship between RAI dose and RAI related symptoms frequency. Consistent with our findings, Schlumberger et al. also found comparable frequency of RAI related symptoms between low and high RAI doses administered after thyroid hormone withdrawal (THW) [20]. In contrast, Grewal et al. and Mallick et al. found a direct relationship between RAI related symptoms frequencies and RAI dose [13, 21].

After a thorough review of the literature regarding RAI related symptoms, we could not find a validated instrument to evaluate their frequency and intensity. The lack of this tool motivated us to develop a web-based survey to evaluate the presence of early and late RAI related symptoms, including the 12 most frequently published in previous studies [12, 13, 15, 16]. The aim of these surveys was to study both the frequency and intensity of these RAI related symptoms.

The frequency of RAI related symptoms reported by patients who did not receive RAI was unexpectedly high. One plausible explanation could be the effect of hypothyroidism required before RAI administration. As reported by Mallick et al. and Schlumberger et al. in HiLo and ESTIMABL trials, respectively, patients who received RAI with THW had a higher frequency of RAI related symptoms compared to those with rhTSH administration [20, 21]. Some of the RAI related symptoms they found, such as “puffy face” or “weight gain,” could be considered as equivalent to some of the symptoms reported by our cohort. This could also explain, to some extent, the higher frequency of early RAI related symptoms in those who received RAI (with THW) compared to those who did not. However, this higher frequency persisted in late RAI related symptoms when all patients were under thyroid hormone replacement. This fact verifies that the influence of RAI administration in the frequency of RAI related symptoms is not entirely dependent on the hypothyroid state.

Another explanation for the high frequency of RAI related symptoms reported by patients who did not receive RAI is potential selection bias of the study, considering that nearly half of patients answered both surveys. Further, these patients may have been more prone to report symptoms than those who did not accept our invitation and may be especially willing to interpret any discomfort as a positive outcome. Nonetheless, there were significant differences between those who received RAI and those who did not, confirming the validity of our survey. Currently, our group is prospectively surveying all patients who receive RAI in our center. This will allow us to increase the number of analyzed patients, avoid bias, and validate our results.

Other relevant adverse effects have been reported in the literature for the use of RAI. There is an increased risk of secondary malignancies, including solid tumors and leukemia, even in patients with low risk thyroid cancer that have received RAI [9, 10]. These issues are beyond the scope of our study, but they should also be considered when defining the need of RAI ablation in patients with PTC, thus restricting its use only in patients who will get a real benefit [9].

Recent publications have shown important effects of DTC on QoL [22, 23]. A recent publication by Applewhite et al. has shown that QoL scores among thyroid cancer patients were the lowest at initial diagnosis, surgery, THW, and, interestingly, RAI ablation [22]. When compared to patients affected by other neoplastic diseases with poorer survival, thyroid cancer patients showed similar QoL scores. This fact reveals that QoL impairment is defined not only by prognosis but, probably, by the intrinsic nature of the disease and some treatment related factors. Hence, in order to reduce distress associated with DTC diagnosis and treatment, we should be cautious when deciding therapeutic approaches, weighting benefits and physical and psychological side effects [24]. Probably, RAI is a paradigm of a therapy with questionable benefit and evident side effects in low risk DTC patients.

In conclusion, this study shows a high frequency of early and late RAI related symptoms in patients with DTC, even when low doses are used. This information should be carefully considered when defining what dose to administer, especially among patients with low risk of recurrence, since an improvement on prognosis is questionable.

## Supplementary Material

Radioactive Iodine (RAI) Related Symptoms Survey

## Figures and Tables

**Table 1:** Demographics, clinical features, and risk stratification of studied patients.

	Patients who answered only early survey	Patients who answered both surveys	*p*
*Participants (n*)	110	61	
*Females (%)*	91,8	96,7	ns
*Age at diagnosis (years ± SD)*	43,4 ± 13,8	41,4 ± 14,5	ns
*Type of surgery*			
Total thyroidectomy (TT) (%)	76,4	68,9	ns
TT + lymph node Resection (%)	23,6	31,1	ns
*ATA classification at diagnosis*			
Low risk (%)	54,5	52,5	ns
Intermediate risk (%)	45,5	47,5	ns
High risk (%)	0	0	ns
*Time to survey*			
First survey (months ± SD)	2.5 (0.5–5.8)		na
Second survey (months ± SD)	—	11.5 (6–18.6)	na
*Radioiodine therapy*			
Patients treated with RAI (%)	78,2	80,3	ns
Median RAI dosage (range)	100 (30–150)	100 (30–150)	ns
ATA low risk patients with RAI (%)	63,3	62,5	ns
ATA intermediate risk patients with RAI (%)	96	100	ns
