# Supplementary material for: Radioactive Iodine Administration Is Associated with Persistent Related Symptoms in Patients with Differentiated Thyroid Cancer
Source: Int J Endocrinol. 2016 Oct 27;2016:2586512. doi: 10.1155/2016/2586512 (PMC5102728; doi:10.1155/2016/2586512)
Supplement: Supplementary file 1 — Radioactive Iodine (RAI) Related Symptoms Survey [file 2586512.f1.docx]

**SUPPLEMENTARY TABLE 1: SURVEY**

**RADIOACTIVE IODINE (RAI) RELATED SYMPTOMS SURVEY**

RAI therapy preparation: Thyroid hormone withdrawal____ Intramuscular rhTSH ____

After RAI administration, have you had any of the symptoms listed below? If your answer is yes, please rate the intensity of the symptoms and state the time of appearance after RAI administration. If you have not received RAI, please answer considering time of surgery.
*(in italics the Spanish version of the question)*

|  | **Symptom** | **No** | | **Yes** (please rate the intensity of the symptom: 1 very mild; 2 mild; 3 moderate; 4 intense; 5 very intense) | | | | | | | |
| --- | --- | --- | --- | --- | --- | --- | --- | --- | --- | --- | --- |
|  |  |  |  | 1 | | 2 | | 3 | | 4 | 5 |
| 1 | **Puffiness around the eyes**  *(Hinchazón alrededor de los ojos)* |  | |  | |  | |  | |  |  |
|  | Days after treatment |  | | | | | | | | | |
| 2 | **Dry eyes**  *(Sensación de ojo seco)* |  | |  | |  | |  | |  |  |
|  | Days after treatment |  | | | | | | | | | |
| 3 | **Excessive tearing from one or both eyes** *(Lagrimeo de uno o ambos ojos)* |  | |  | |  | |  | |  |  |
|  | Days after treatment |  | | | | | | | | | |
| 4 | **Conjunctivitis: red eyes or swelling**  *(Conjuntivitis: ojo rojo, hinchazón)* |  | |  | |  | |  | |  |  |
|  | Days after treatment |  | | | | | | | | | |
| 5 | **Pain around salivary glands: cheeks, in front of the ears, below the mouth**  *(Dolor en la zona de glándulas salivales: mejillas, delante de las orejas y bajo la boca)* |  | |  | |  | |  | |  |  |
|  | Days after treatment |  | | | | | | | | | |
| 6 | **Swelling around salivary glands: cheeks, in front of the ears, below the mouth**  *(Aumento de volumen en la zona de las glándulas salivales: mejillas, delante de las orejas y bajo la boca)* |  |  | |  | |  | |  | |  |
|  | Days after treaatment |  | | | | | | | | | |
| 7 | **Dry mouth**  *(Sensación de boca seca)* |  | |  | |  | |  | |  |  |
|  | Days after treatment |  | | | | | | | | | |
| 8 | **Loss or impairment in sense of taste**  *(Pérdida o disminución del sentido del gusto)* |  | |  | |  | |  | |  |  |
|  | Days after treatment |  | | | | | | | | | |
| 9 | **Changes in quality of taste**  *(Cambio en el gusto de las comidas)* |  | |  | |  | |  | |  |  |
|  | Days after treatment |  | | | | | | | | | |
| 10 | **Loss or impairment in sense of smell**  *(Pérdida o alteración del olfato)* |  | |  | |  | |  | |  |  |
|  | Days after treatment |  | | | | | | | | | |
| 11 | **Pain in cervical area (neck)**  *(Dolor en la región cervical [cuello])* |  | |  | |  | |  | |  |  |
|  | Days after treatment |  | | | | | | | | | |
| 12 | **Nausea**  *(Náuseas)* |  | |  | |  | |  | |  |  |
|  | Days after treatment |  | | | | | | | | | |
| 13 | **Vomiting**  *(Vómitos)* |  | |  | |  | |  | |  |  |
|  | Days after treatment |  | | | | | | | | | |
